# Perception of the usability and implementation of a metacognitive mnemonic to check cognitive errors in clinical setting

**DOI:** 10.1186/s12909-018-1451-4

**Published:** 2019-01-10

**Authors:** Keng Sheng Chew, Jeroen J. G. van Merrienboer, Steven J. Durning

**Affiliations:** 1 Faculty of Medicine and Health Sciences, Universiti Malaysia Sarawak, Kota Samarahan, Malaysia; 2 School of Health Education, Maastricht University, Maastricht, The Netherlands; 3 Uniformed Services University of the Health Sciences, Bethesda, USA

**Keywords:** Cognitive errors, Clinical decision making, Mnemonic, Checklist, Usability, Implementation

## Abstract

**Background:**

Establishing a diagnosis is a complex, iterative process involving patient data gathering, integration and interpretation. Premature closure is a fallacious cognitive tendency of closing the diagnostic process before sufficient data have been gathered. A proposed strategy to minimize premature closure is the use of a checklist to trigger metacognition (the process of monitoring one’s own thinking). A number of studies have suggested the effectiveness of this strategy in classroom settings. This qualitative study examined the perception of usability of a metacognitive mnemonic checklist called TWED checklist (where the letter “T = Threat”, “W = What if I am wrong? What else?”, “E = Evidence” and “D = Dispositional influence”) in a real clinical setting.

**Method:**

Two categories of participants, i.e., medical doctors (*n* = 11) and final year medical students (Group 1, *n* = 5; Group 2, *n* = 10) participated in four separate focus group discussions. Nielsen’s 5 dimensions of usability (i.e. learnability, effectiveness, memorability, errors, and satisfaction) and Pentland’s narrative network were adapted as the framework to study the usability and the implementation of the checklist in a real clinical setting respectively.

**Results:**

Both categories (medical doctors and medical students) of participants found that the TWED checklist was easy to learn and effective in promoting metacognition. For medical student participants, items “T” and “W” were believed to be the two most useful aspects of the checklist, whereas for the doctor participants, it was item “D”. Regarding its implementation, item “T” was applied iteratively, items “W” and “E” were applied when the outcomes did not turn out as expected, and item “D” was applied infrequently. The one checkpoint where all four items were applied was after the initial history taking and physical examination had been performed to generate the initial clinical impression.

**Conclusion:**

A metacognitive checklist aimed to check cognitive errors may be a useful tool that can be implemented in the real clinical setting.

**Electronic supplementary material:**

The online version of this article (10.1186/s12909-018-1451-4) contains supplementary material, which is available to authorized users.

## Background

An overarching concern a doctor faces in generating differential diagnoses is whether enough data have been gathered to make a diagnosis. Failure to do so may lead to fallacious cognitive tendencies such as premature closure, which may in turn, lead to diagnostic errors [1]. Premature closure refers to the tendency to close the diagnoses generation process before a diagnosis has been fully verified [2].

Differential diagnoses generation is typically believed to be a complex, iterative process involving two inter-related steps [3, 4]. The first step (*hypothesis generation*) is predominantly a fast, non-analytical step construed through a pattern-recognition process. It works by matching the patient’s presenting data with the doctor’s mental disease models (also called ‘illness scripts’ or ‘schemas’) [3, 4]. In dual-process theory, this step is known as a Type 1 thinking process [5, 6]. The second step, known as *hypothesis evaluation*, is predominantly a slower, analytical process of evaluating competing diagnoses prompted by the mental representations in the first step. In dual-process theory, this is known as a Type 2 thinking process [5, 6]. These two processes interact with one another (e.g., the analytical evaluation of competing diagnoses alters the mental representation of the patient data and vice versa), until a most probable diagnosis is reached [5, 6]. The failure to gather sufficient data may result in premature closure. For example, if one misses the history of sympathomimetic substance abuse such as cocaine in a profusely diaphoretic patient with chest discomfort and elevated body temperature, one might miss considering cocaine-induced myocardial ischemia as a potential diagnosis [7].

A number of strategies have been proposed to minimize cognitive errors like premature closure [8, 9]. As premature closure is believed to be more prevalent in Type 1 thinking processes than in Type 2 thinking processes [1, 10], a proposed strategy to mitigate premature closure is metacognitive monitoring, i.e., a process of critical self-reflection by cognitively slowing down and monitoring one’s own thinking [11–13]. In this regard, a metacognitive checklist functions as an activation trigger in facilitating the “slow thinking” process of reflecting on the plausibility of one’s diagnostic workup [14–17].

For example, in a study on the effectiveness of a checklist meant to induce reflection on diagnostic reasoning after making an initial diagnosis, it was found that metacognitive monitoring results in more accurate diagnoses, particularly for complex or unusual clinical cases [14]. As another example, in a study conducted with a group of final year medical students using a mnemonic checklist called the TWED checklist (where the letter “T = Threat”, “W = What if I am wrong? What else?”, “E = Evidence,” and “D = Dispositional influence”), it was shown that this checklist was effective in helping the task performer generate more relevant differential diagnoses [18, 19]. Nonetheless, from a non-exhaustive focused search by author KSC using Web of Science, MEDLINE and Google Scholar, most studies on the use of non-mnemonic checklists to facilitate metacognitive monitoring [14, 15, 17–21] have been conducted using paper-based assessments in classroom settings. The present study will investigate the use of a metacognitive checklist in a real clinical setting and consider its usability and implementation in work routines.

According to Nielsen (1996), it is often simplistic to assume that the usability of a tool is unidimensional [22]. Instead, he proposed a multi-dimensional metric originally meant to assess the usability of a tool in a human-computer interface context [21]. The five dimensions of the Nielsen’s usability metric are: (1) learnability of the tool – how easy is it for users to accomplish the intended task the first time they are using it? (2) effectiveness – once users have learned the design, how quickly and effectively can they perform the intended tasks using the tool? (3) memorability – when users return to the tool after a period of not using it, how easy can they reestablish proficiency in using the tool? How easy is it for them to remember the characteristics of the tool? (4) errors/pitfalls – what are the errors, pitfalls or flaws in using the tool? (5) satisfaction or pleasantness – how satisfied or pleasant is it to use the tool [22]?

Besides its usability, how well a checklist can be implemented in a work routine should also be considered. In this regard, a narrative network can be a helpful framework in mapping out the potential new patterns of actions that can be implemented in a routine work process [23]. An example described by Pentland and Feldman (2007) is the mapping of the various permutations of implementing information and communication technology (ICT) in airline ticket purchase [23].

Although previous studies [18, 19] have suggested the usefulness of the TWED checklist in reducing the risk of cognitive errors, these studies were conducted in classroom settings. Making clinical decisions in a classroom setting falls short of the ecological validity of a busy clinical ward. This is because in a classroom setting, one could possibly focus fully on working on the case scenarios without being distracted by the “multi-sensorial” noises as expected in a busy ward (e.g., sight, sound, smell, the competing demands, the chaotic work nature, etc. as expected in an emergency department). This study intends to fill a gap in the literature by addressing two research questions: (1) how useful is a checklist that facilitates metacognitive regulation in differential diagnoses generation in such real clinical environment? And (2) how can users implement such a checklist in their daily clinical routines? To answer the first research question, the five dimensions of usability based on Nielsen (1996)‘s metric (i.e., learnable, memorable, effective, pleasant, as well as potential pitfalls or limitations) [22] is used as the conceptual framework whilst to explore the second question on the various ways that a metacognitive checklist can be implemented in the complex process of differential diagnoses generation, the narrative network [23] was adopted.

## Method

To capture the perception on how doctors and medical students interacted with the TWED checklist in actual clinical settings, the two research questions mentioned above were answered qualitatively by means of focus group discussions [24].

### Participants

Two categories of participants were purposively sampled in this study. The first category consisted of medical doctors with at least 3 years’ clinical experience from the emergency and trauma department of Sarawak General Hospital, Malaysia. Eleven doctors (4 male, 7 female) with ages ranging from 28 to 32 years old were recruited. Medical doctors with less than 3 years of clinical experience as well as doctors who are still undergoing supervised internship were excluded. One participant who initially agreed to participate had subsequently withdrawn due to work commitments. Responses from this group of participants were coded to answer both the first and second research questions.

The second category of participants consisted of final year medical students from the faculty of medicine and health sciences of Universiti Malaysia Sarawak (UNIMAS). The medical degree program of UNIMAS is a 5-year medical program where all final year medical students must have successfully completed and met the passing criteria for year-4 study before they can be promoted to their final year of study. As these students are not yet licensed medical practitioners, responses from this group of participants were coded to answer the first research question only. Thus, questions related to the implementation of the checklist in the clinical setting (i.e., the second research question) were not raised in this group. Two separate focus groups were organized from this category: medical student group 1 consisted of 5 students (2 male and 3 female) aged between 24 and 25 years old, and the medical student group 2 consisted of 10 students (1 male and 9 female) aged between 24 and 25 years old.

A total of four focus group discussions were conducted (one session each for the medical student group 1 and medical student group 2, and two sessions for the doctor group). For both medical student groups, only one session was held per group due to logistic challenges as the students had to undergo a tight schedule in completing the various clinical rotations. All participations were volitional, and no monetary compensation was involved in the recruitment of the participants.

### Materials

Prior explanation on diagnostic errors, premature closure and other types of cognitive errors in clinical decision-making as well as the application of the metacognitive checklist (i.e., TWED checklist) was given to participants from both the medical students and doctor groups three months before starting the focus group discussions.

The TWED mnemonic checklist is a 4-item checklist purported to minimize the tendency of cognitive errors such as premature closure [25]. The first item represented by the letter “T = Threat” is to reflect on the question “Is there any life or limb threatening conditions I need to rule out in this patient?” The second item, “W = What else?” is about reflecting on the questions of “What if I am wrong? What else?” The third item “E = Evidence” is about reflecting on the question of the sufficiency of the evidence or data to support or refute a particular diagnosis (“Do I have enough evidence to support or exclude this diagnosis?”) whereas the fourth item, “D = Dispositional influences” deals with the hidden emotional or environmental dispositional factors (e.g. doctor’s fatigue, busy emergency ward, etc.) that may influence the quality of the diagnostic decisions. Hence, reflecting on the first two items (“T = Threat”, “W = What else?”) may trigger more patient data collection, whereas reflecting on the third item (“E = Evidence”) may trigger the evaluation of how well a diagnosis under consideration is supported by the various pieces of patient data. The fourth item (“D = Dispositional influence”) acts as an overarching self-reflective mechanism to guard against premature closure due to extrinsic influences [25].

During the focus group discussions, open questions on the five dimensions of usability (i.e. learnability, effectiveness, memorability, errors, and satisfaction) [22] were based on the following scheme:

1. How easy was it for you to learn how to use this checklist in your clinical encounters with the patients (“learnability”)? Explain your response.
2. How effective do you think this checklist is in preventing diagnostic errors or near-missed diagnoses (“effectiveness”)? Do you have any specific examples to share? Explain your response.
3. How easy was it for you to remember the items or components of this checklist, especially after a period of not using it (“memorability”)? Explain your response.
4. Have you encountered any flaws, errors, limitations or pitfalls while using this checklist (“pitfalls/limitations”)? Explain your response.
5. How satisfied were you with using this checklist in clinical decision making (“satisfaction”)? Explain your response.

Regarding the narrative network used to answer the second research question, the narrative or the steps of how differential diagnoses generation would routinely occur (“pre-implementation”) was first constructed. This was accomplished through group discussion (by participants in the doctor group) and differences of opinion were resolved through working towards general consensus during the focus group discussions. In the unlikely event that consensus could not be reached, one of the authors (KSC) would make a final decision after taking all opinions into consideration. The various permutations on how the various components of the TWED checklist were implemented into the routines of differential diagnoses generation was then discussed in order to construct the “post-implementation” narrative.

### Procedure

Explanations on different types of diagnostic errors, classification of cognitive errors in clinical decision-making as well as on the application of the TWED checklist were given by one of the authors (KSC) to all participants three months prior to the commencement of the focus group discussions. Participants were given a digital copy of the TWED checklist, delivered to their smartphones and electronic devices. For the next three months, they were told to use the checklist as often as they wanted to, and in any manner deemed suitable. At the end of the three months, focus group discussions were held.

Participants were told that their discussions would be audio-recorded and transcribed anonymously (i.e., their identities would not be revealed and that they would be identified in such manner as Student 1, Student 2, etc. for the medical student groups or as Doctor 1, Doctor 2, etc. for the doctor group). They were also assured of the confidentiality of their responses.

As mentioned, for the doctor group only, the focus group discussions were slightly longer (2 h versus 45 min to one hour in the medical student focus group discussions). This was because after the discussion related to the first research question on the usability of the checklist, participants in this group were also asked how they implemented the checklist in their clinical practice. All focus group discussions were conducted in classrooms with participants seated facing each other in circular arrangement.

One of the participants in each group was selected as the moderator of the group prior to starting the first session of the focus group discussions. The moderator was first briefed and given the interview scheme on the usability of the checklist as well as the framework for the narrative network. One of the authors (KSC) acted as the note-taker to scribble down the participants’ responses, observed the dynamics of the group interactions as well as to ensure that the discussions stayed in focus.

Transcriptions of the audio recordings were performed by one of the authors (KSC). The transcripts were then sent back to the participants for member-checking. This author (KSC) and another independent medical doctor (who was not a co-researcher of this study) from Sarawak General Hospital then performed the open coding deductively using thematic content analysis method through iterative readings and labelling of keywords and phrases from these transcripts [26]. After the initial open coding, a second axial coding was performed by re-analyzing these open codes to look for patterns and relationships among them. Discrepancies between the codings by the two coders were resolved through face-to-face and online discussions. NVivo version 12.0 for Mac software was used to aid the coding process. Approval from the grants and research ethics committee of UNIMAS was obtained prior to commencement of the study (F05/SoTL/1477/2016).

## Results

### The perception of checklist usefulness

To answer the first research question on the perception of usability of the checklist in clinical setting, themes identified along with their illustrative comments, anecdotes and how these themes can be related to the TWED checklist are compiled and tabulated in detail in Table 1. With regards to the dimension of effectiveness, this checklist was perceived to be effective in promoting metacognition. This finding is probably best encapsulated in the below quote:

**Table 1:** Key themes identified (first phrase in bracket refers to the group the participant belonged, second phrase refers to the anonymized identity of the participant)

Dimensions of usability metric (Nielsen’s 1996)	Themes	How it relates to TWED checklist and illustrative anecdotes
Learnability	Learnability is believed to be facilitated should the checklist is introduced early. Illustrative remarks: *“Yeah, this tool should be taught earlier... as early as possible, maybe while in medical school because once they already out working, the junior doctors would have already developed their own ways of approaching patients, and it might be difficult to introduce new tools for them” (Doctor 10)* *“…this checklist would be useful but it should be introduced much earlier at the beginning of our clinical rotations in Year 3; then we would be more familiar with it.” (Student 4, Group 1)*	
	Learnability can be hampered by lack of emphasis on critical thinking in medical school Illustrative remarks: *“Yes, it takes some time for us to learn this checklist, I mean, this is something new to us, and it is because I think many of our lecturers have seldom been emphasizing on questioning the rationale behind why we choose this working diagnosis.” (Student 1, Group 1)*	
	Its simplicity makes the checklist easy to learn Illustrative remarks:s *“The checklist is not that complicated to learn with only four items to it” (Student 1, Group 2)*	
Effectiveness of the checklist	Effectiveness in resolving diagnostic dilemma It is an effective tool in helping to resolve decision dilemmas, particularly, whether to discharge apparently stable patients with non-specific complaints. Illustrative remarks: *“…this tool is very helpful in evaluating patients in the Green zone. This is because of the volume of patients that come into the Green zone, especially for patients that come in with very nonspecific complaints. So, by just applying this checklist, it can help us to rule out the life threatening causes for these nonspecific complaints…” (Doctor 9)*	Item W = “What else?” should especially be activated if there are data that does not fit into the overall clinical picture of the patient. Illustrative anecdote: *“I asked myself, why was the patient on wheelchair? He does not need to be on wheelchair if he just had URTI. How long has he been on wheelchair? Is it because he’s not able to walk by himself? And if he’s not able to walk, how long has he been in this state? Then I asked myself, “What else could this be then?” And then I asked the family members, “Why is he on a wheelchair? Has he always been on wheelchair, not able to walk on his own?” It was only then that the family member said, “Oh, I am sorry. I forgot to inform you just now. He suddenly could not walk anymore. Just about 2 h ago.” (Doctor 2)*
	Effectiveness in promoting metacognition It is also an efficient tool in promoting metacognition. Illustrative remarks: *“This tool will at least help me not to simply discharge the patient, reducing my risk of misdiagnosing and mismanaging the patient.” (Doctor 10)*	Parental anxiety should prompt the doctor to be extra careful in considering the question of “What else?” Illustrative anecdote: *“So, initially I thought of discharging this patient who looked so well but ehh… at that time, I applied this tool and asked myself what else I could have missed given the unusual presentation of left knee pain. Furthermore, the parents were quite worried. So, I decided to do an x-ray of for him and lo and behold, I’ve found out that this pain is due osteochondroma.” (Doctor 10)* Item E is especially important in bringing more objectivity to the diagnostic process and to counter authority gradient in diagnoses consideration. Illustrative anecdote: *“I remember a patient I encountered. Every clinician said that was the case of Guillain-Barre syndrome but it turns out not to be so. Err. it was likely because initially the visiting neurologist told every clinician at that time that the case was Guillain-Barre syndrome. But actually when I elicited the reflexes, I found it to be rather brisk. So I went back to the checklist and ask myself “what else could it be?” And I began to look hard for other evidence. Eventually after some other investigations done by the doctors in charge, it turned out that the patient actually had mononeuritis multiplex.” (Student 1, Group 1)*
Memorability	Relevance of checklist determines its memorability Illustrative remarks: *“…generally the contents of this tool are exactly what we do in our day-to-day clinical work. Basically when we see patients, no matter which zones we are working in, the first thing is to rule out life threatening causes; only then we start ruling out all other possible diagnoses, and only then we make decisions on whether to admit or to discharge the patients. So, basically, thought processes embedded in the tool are very simple and generic…” (Doctor 5)*	Items “T” (Ruling out life or limb threatening conditions) and “W” (What if I am wrong? what else?”) are the most important/useful components of the checklist to both medical students and the doctors and is the only item that some medical students can remember. Illustrative remarks: *“..first two items (*i.e.*, the items “T” and “W”), they are easier for us to remember because these two items are relevant to us in our clinical encounters as medical students, because we apply them everyday. The other components or items are more difficult to remember.” (Student 2, Group 1)* *“Parts of this checklist are user friendly; but other parts are not. Like for example, item no. 1, the “T”. and item no. 2, “W”, “Is there any life threat?” “What if I am wrong”, the words themselves are self-explanatory. I know what it is about. But item no. 4 “dispositional factors” is complicated. I wouldn’t understand what it means and I would have to read further on the fine prints to understand it. And in an emergency situation, I wouldn’t be able to do that.” (Student 5, Group 1)* items “W” and “E” are inter-related as the consideration of one of these 2 items may trigger the consideration of the other item. Illustrative remarks: *“In my opinion, I think after we consider ‘E = evidence’, we should go back to ‘W’,* i.e.*, whether we are wrong or not? Or whether the evidence support my diagnoses or not? And what else it could be? (Student 5, Group 1)”* *“…I think that there are some overlaps between item no. 2 (“W”) with item no. 3 (“E”). Because, by the fact that I can say I might be wrong means that I have evidence to show it to be so…” (Student 1, Group 1)*
	Familiarity of the checklist determines its memorability The items in the checklist where the participants can remember are the items that they are familiar with. Ironically however, familiar items are precisely the items that they have been practicing in their daily clinical work, hence, many believe they do not need a checklist for these processes. Illustrative remarks: *For me, I think the components “T”, “W”, “E” are something which we are already practicing on a daily basis even without referring to the checklist but the “D = Disposition” component of the tool is something we need to pay particular attention too. (Doctor 9)*	Most of the medical students perceive “item D” (the dispositional influence of emotional and environmental factors on the clinical decisions) as not applicable, not important and not relevant. Illustrative remarks: *“I don’t know… I just remember the item “T”, to rule out the emergency conditions. (Student 2 nodded in agreement). The other items “E = Evidence” and “D = the Dispositional factors” do not occur to me as relevant most of the time.” (Student 1, Group 1)* On the contrary as illustrated in the remark in the column on the left, most of the doctors can relate on the importance of “item D” as an essential but often neglected group of factors influencing the quality of their clinical decisions. Illustrative remarks: *“…I think the components “T”, “W”, “E” are something which we are already practicing on the daily basis but the “D = Disposition” component of the tool is something we need to pay particular attention too. The “environment” we are working in can influence our judgment.” (Doctor 9)*
Errors/Pitfalls/limitations	The checklist may slow down the entire working process Another limitation of the checklist is that it can be time-consuming and slow down the entire work process, especially in a busy clinical setting. Illustrative remarks: *“To me the limitation of this tool is that when we keep thinking too much on the patient, we may then be worrying too much about the case, spending too much time thinking of what could the errors be,* etc. *I mean, being a bit skeptical, applying critical thinking is good, but sometimes, this can become too time-consuming especially when we are too skeptical, which in turn, delays our management, leading to stress and frustration and prolonging the waiting time for the patients. This, I think is particularly true for cases that are stable or relatively stable; in Green zone, for example, although, I mean, this tool would be helpful for patients with unstable vital signs, but for patients who are stable in Green zone, I think, applying this tool is too time consuming. In other words, where the diagnosis is clear cut, I would probably not likely to apply the tool, but where the diagnosis is not clear cut, but I know something is not right the patient, I would probably apply it. The challenge for us then is to know when and for which case do we need to apply the checklist, and which ones we do not.” (Doctor 8)*	
	The checklist requires adequate prior medical knowledge. Hence, the effectiveness of the checklist is hampered by the lack of prior medical knowledge. In fact, as one student put it, this checklist is not pleasant to use because it reminds him of his own inadequacies: *“I believe the challenge in using this checklist is contributed by our own lack of knowledge. It is mostly because of our own inadequacies; for example, we can usually only think of 3–4 differential diagnoses.” (Student 1, Group 2)*	Only one student agreed that item “D” is relevant, but as he has rightly said (see remarks below), even if students are aware of their own fallacy, they may feel helpless as their knowledge base is inadequate for them to generate another differential diagnosis: *“I think the item “D” is still relevant to us as medical students. Since we are humans, our judgment can also be influenced by the emotional state that we are in. But the real problem is, I think, even if we know the emotional and environment dispositions that may influence our judgment, often we are still not able to generate alternative diagnoses due to the lack of knowledge.” (Student 3, Group 2)* On the other hand, some perceived the checklist as an unpleasant tool to use as it mirrors their own inadequacies: *“To me, this tool is not pleasant to use. It is supposed to be a checklist, but to me this is too complicated. Maybe because I am not familiar with it. I think there are just many items in it. I cannot remember all. I think it is supposed to be simpler than this.” (Student 2, Group 2)*
Satisfaction or pleasantness in using the checklist	Its mnemonic and simplicity makes it pleasant to use Illustrative remarks: *it is pleasant to use this checklist with its mnemonic structure and also because, since it only has four items, it is simple enough (Student 2, Group 2)*	
	The fact that it reminds the participant of his or her own inadequacies and shortcomings (of not able to generate more differential diagnoses) makes it unpleasant to use Illustrative remarks: *“Actually I think this checklist is not very pleasant to use in the sense that it reminds me of my own inadequacies and shortcomings but it is still a good checklist to use to help us remember to check for things we might have missed.” (Student 3, Group 2)*	

Note: (first phrase in bracket refers to the group the participant belonged, second phrase refers to the anonymized identity of the participant)

*“This tool will at least help me not to simply discharge the patient, reducing my risk of misdiagnosing and mismanaging the patient.” (Doctor 10).*

However, it seems that not all four items in the TWED checklist are equally memorable. The participants from both categories commented on the fact that the memorability of a checklist is not just dependent on its organization and mnemonic structure but also on its relevance and familiarity. Participants from both categories could remember to apply items “T” (threats) and “W” (what else) as these two items are important and directly relevant to their clinical practices. But, most participants in the medical student groups could not remember the items “E” (evidence) and “D” (dispositional influence). They perceived these two items as not relevant and important. This is because these medical students are seeing patients for the purpose of learning, and not for the purpose of actually treating or managing them. This is typified in the following two quotes:

*“I think for the first two items (i.e., the items “T” and “W”), they are easier for us to remember because these two items are relevant to us in our clinical encounters as medical students, because we apply them everyday. The other components or items are more difficult to remember.” (Student 2, Group 1).*

*“I can only remember one item out of the four; and that is the item T which stands for life-threatening conditions, because that is the most important thing for us, ruling out emergency conditions. For example, when I encountered a case of antepartum hemorrhage, one of the first things I must think of is abruptio placenta because that is an emergency.” (Student 4, Group 2).*

Some participants in the medical student group also commented that items “W” and “E” are inter-related as the consideration of one of these 2 items may trigger the consideration of the other item. For example,

*“In my opinion, I think after we consider ‘E = evidence’, we should go back to ‘W’, i.e., whether we are wrong or not? Or whether the evidence support my diagnoses or not? And what else it could be? (Student 5, Group 1)”.*

*“…I think that there are some overlaps between item no. 2 (“W”) with item no. 3 (“E”). Because, by the fact that I can say I might be wrong means that I have evidence to show it to be so…” (Student 1, Group 1).*

On the other hand, a number of participants in the doctor group commented that items “T”, “W” and “E” are items that they are already familiar enough with and have been practicing unconsciously on a daily basis. Hence, they felt that they do not need a checklist for these three items. On the contrary, unlike the medical students, for these doctors, item “D = Disposition” represents an often-neglected but relevant group of factors. Hence, they felt that they needed to pay more attention to how these factors influence the quality of their diagnostic decisions. This is illustrated by the quote below:

*“…I think the components “T”, “W”, “E” are something which we are already practicing on the daily basis but the “D = Disposition” component of the tool is something we need to pay particular attention too. The “environment” we are working in can influence our judgment.” (Doctor 9).*

Third, in terms of its learnability, the participants believed that as the checklist only has four items, it is not complicated to learn. In order to enhance the learning of such this checklist, participants from both categories agreed that it should be introduced as early as possible in medical schools, preferably before a medical student begins his or her clinical rotations. For example, one of the participants in the doctor group commented:

*“Yeah, this tool should be taught earlier… as early as possible, maybe while in medical school because once they already out working, the junior doctors would have already developed their own ways of approaching patients, and it might be difficult to introduce new tools for them” (Doctor 10).*

Fourth, in terms of the dimension of limitations or pitfalls of the checklist (i.e., limitations), participants from both categories believed that the effectiveness of the checklist is hampered by a lack of prior medical knowledge. In other words, although this checklist may be effective in promoting metacognition, it is not a “magic bullet” in resolving premature closure. For it to work, the doctors and medical students need to have adequate prior knowledge. Another limitation highlighted by the participants from the doctor group was the fact that applying this checklist on every case can be time consuming; hence, it may slow down the entire working process. This is illustrated by the quote below:

*“To me the limitation of this tool is that when we keep thinking too much on the patient, we may then be worrying too much about the case, spending too much time thinking of what could the errors be, etc. I mean, being a bit skeptical, applying critical thinking is good, but sometimes, this can become too time-consuming especially when we are too skeptical, which in turn, delays our management, leading to stress and frustration…. The challenge for us then is to know when and for which case do we need to apply the checklist, and which ones we do not.” (Doctor 8).*

And lastly, regarding the satisfaction or pleasantness in using the checklist (i.e., satisfaction), although some participants believed that its mnemonic and simplicity makes the checklist pleasant to use, there are also participants who commented that as the checklist exposed them to their own shortcomings (i.e., inadequate prior knowledge) in generating pertinent different diagnoses, hence, the checklist is in not so pleasant to use.

### Implementation of checklist

**Fig. 1:**
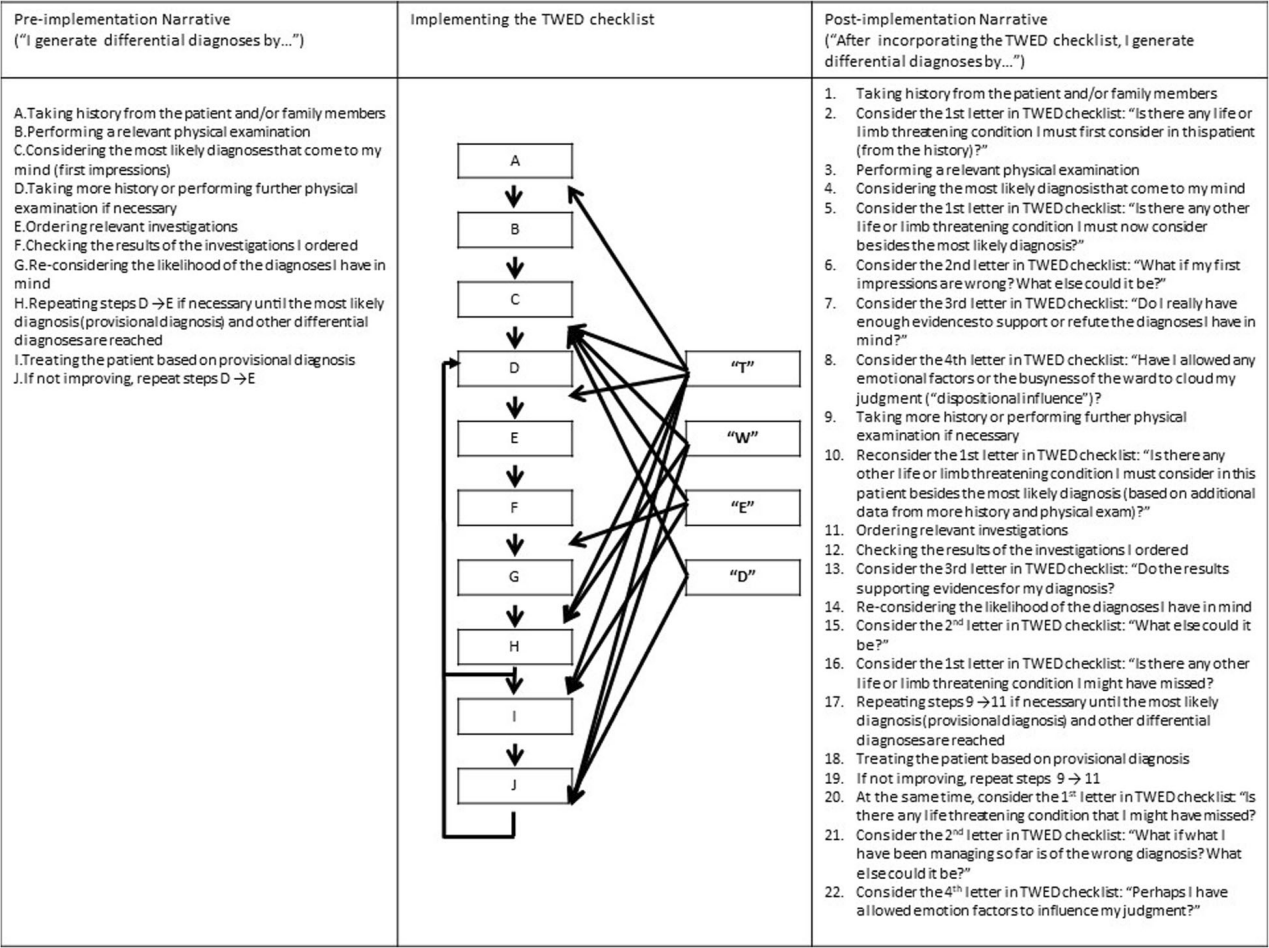
showing the narrative network on how the specific items of TWED checklist can be implemented in the diagnostic process

To answer the second research question, a narrative network was constructed. In this narrative network, the narrative of the diagnostic process prior to implementation of the TWED checklist (i.e., pre-implementation narrative) was mapped with the specific items in the checklist in order to generate the so-called post-implementation narrative (see Fig. 1). From the post-implementation narrative, it is apparent that the items in the checklist are not applied in the same manner and/or with the same frequency. The item “T = Threat” was applied iteratively at almost every cognitive checkpoint when decisions are to be made, for example, after taking history and performing physical examination, after obtaining further data from more history-taking and re-examining the patient, when reconsidering differential diagnoses based on data from investigations, or when the patient is not improving as he or she should be. On the other hand, the participants believed that the items “W = What if I am wrong? What else?” and “E = Evidence” should only be applied when the outcome did not appear the way that it should be, for instance, when the patient is not improving. Finally, the “D = Dispositional influences” is the item that needs to be applied infrequently. It is akin to a “cognitive brake” in the fast lane of clinical decision making, to ensure that one does not allow emotional influences or the ambient atmosphere of the workplace environment to cloud or distort one’s judgment. The one cognitive point where all four items converge is after the initial history taking and physical examination has been performed to generate the initial clinical impression (Step C in the pre-implementation narrative as shown in Fig. 1).

## Discussion

With regard to the first research question, this qualitative study suggests that the TWED checklist is a useful tool to both medical students and doctors. As commented by a participant in the student group, by the fact that the checklist only has four items, it is not complicated to learn and should not interfere with the application of other checklists that students have often been taught in medical schools (for example, the “VINDICATED” checklist for etiologies of differential diagnoses where, V = vascular, I = Inflammatory, N = neoplastic, D = drugs, I = Infective, C = congenital, A = autoimmune, T = trauma, E = endocrine and metabolic and D = degenerative). The checklist also appears to be effective in promoting metacognition. However, it can be time consuming, particularly when one first learns how to apply it. In addition, while some participants opined that the mnemonic in the checklist makes it pleasant to use, others felt that it was not. This is due to the fact that the checklist is akin to a mirror in reflecting the inadequacy of their prior knowledge.

Different groups of participants found different aspects of the checklist useful. For the medical students, the items “T = Threat” and “W = What else?” were the most useful. These two items awakened their awareness on the importance of mitigating cognitive errors such as premature closure.

As for the doctors, they claimed that only item “D = Dispositional influences” was something new to them. It helped them realize the importance of emotional and environmental factors in diagnostic decisions. They further claimed that items “T = Threat” and “W = What else?” are practices they had already been doing unconsciously. Excessive and redundant use of checklists could overburden doctors, complicate tasks, and reduce efficiency [27]. This is because, insisting on the use of external tools like a checklist when the clinicians are competent enough may paradoxically tax their working memory, hence, distracting them from the fluent execution of their Type 2 thinking process [28]. The external tool might have triggered the need for the users to reconcile the information contained in the checklist with what they have already internalized [28, 29]. This is further compounded by the fact that the working memory (the active processing memory) is limited in terms of the number of items that can be held at any one time [30, 31]. This potentially counter-productive effect is known as the expertise-reversal effect [28].

On the basis of these findings, while checklist training for novices like medical students should first emphasize on learning items “T” and “W”, the same cannot be said for training doctors. The training for doctors should best be emphasizing on the importance of item “D” only.

With regards to the second research question, the implementation of the TWED checklist in the routine diagnostic process seems simple enough even for the medical students. But just as in learning any new task, integrating this checklist in daily clinical routine takes time and effort. Repetitive practice could allow the tasks embedded in the TWED checklist to be relegated from Type 2 thinking process to the more efficient, automatized Type 1 thinking processes [10].

Some participants from both categories pointed out that the effectiveness of the checklist can be hampered by a lack of prior medical knowledge. The importance of having sufficient knowledge to recognize these cognitive errors and to rectify them with the correct responses has also been alluded to in a number of literature [32–34]. Furthermore, although it is previously mentioned that cognitive errors are believed to be more prevalent in a predominantly Type 1 thinking mode [1, 10], these errors can also occur in a predominantly Type 2 process. As such, merely asking the students and doctors to slow down and reflect on their own thinking using a checklist may not be helpful particularly when they are already slowing down to be more systematic and analytical in their thinking processes (Type 2 thinking) [35]. In other words, implementing the TWED checklist should go in tandem with increasing the knowledge of the participants [36].

In this study, both the Nielsen’s 5 dimensions of usability (i.e. learnability, effectiveness, memorability, errors, and satisfaction) and the Pentland’s narrative network were found to be useful frameworks that can be applied to evaluate the usability and exploring ways of implementation of a psychometric tool in a clinical setting.

A number of limitations of this study warrant mentioning. First, qualitative data were only gathered in one session for each of the two medical student focus groups, and in two sessions for the doctor group. Hence, data saturation was probably not reached for the medical student groups. Second, data were only collected at a single point in time, three months after using the checklist. Had a series of data been collected at different points in time, (e.g., also after 6 months and 9 months), a change of trend might be observed. Allowing a longer period of time for the participants to apply the checklist would afford them the opportunity to master the skill of checking cognitive errors. Ultimately, as the aim of this checklist is to facilitate metacognition to reduce diagnostic errors through minimizing the risk of committing cognitive errors [1], future works could include a quantitative study on the effectiveness of the checklist to reduce the incidence rate of diagnostic errors. However, this could only be done if the doctors have been given sufficient time to master the skill.

Overall, despite its limitations, the results from this study suggest that the TWED checklist is useful in facilitating metacognitive regulation in diagnostic process in a clinical setting. The results imply that this checklist may serve as a trigger to switch from a predominantly automatized decision making mode into a more analytical thinking mode. As aptly stated by Moulton et al. (2017), a good, well-calibrated decision maker is not one who is just able to automatize and speed up a diagnostic process for cases with typical clinical presentations; but one who has mastered the act and the art of “slowing down when one should slow down” [37]. To state it in another way, the critical aspect or the hallmark of a good decision maker is not just the ability to adapt the presenting problems to known illness scripts or clinical solutions but the ability to apply novel or creative solutions to ill-defined or unusual clinical presentations [37]. In Gollwitzer’s conceptual framework [38], the TWED checklist can be said to function as an implementation intention strategy to bridge the gap between the intention of minimizing cognitive errors and the execution of that intended goal.

## Conclusion

Albeit the fact that different groups of participants may use it in slightly different manners (depending on their level of experience), this qualitative study showed that a metacognitive checklist aimed to check cognitive errors is perceived to be likely useful in real clinical setting.

## Additional Files

Additional file 1: Transcript 1 Focus Group Discussion (Group 1 final year medical students) (DOCX 18 kb)

Additional file 2: Transcript 2 Focus Group Discussion (Group 2 final year medical students) (DOCX 16 kb)

Additional file 3: Transcript 3 Focus Groupd Discussion (Doctors Group) (DOCX 24 kb)
