# Supplementary material for: Perception of the usability and implementation of a metacognitive mnemonic to check cognitive errors in clinical setting
Source: BMC Med Educ. 2019 Jan 10;19:18. doi: 10.1186/s12909-018-1451-4 (PMC6327396; doi:10.1186/s12909-018-1451-4)
Supplement: Supplementary file 1 — Transcript 1 Focus Group Discussion (Group 1 final year medical students) (DOCX 18 kb) [file 12909_2018_1451_MOESM1_ESM.docx]

**Focus Group Discussion [Group 1 Final year medical students]**

Student 1/Chairperson: Hi good afternoon. We will start our discussion by focusing on the learnability of this checklist. Anyone has anything to say about this aspect? Do you think it is easy to use?

Student 5: Erm… I think generally it is easy to use; however, sometimes I don't remember to use it.

Student 4: I agree with Student 5, I think it’s because we are not trained from right from Year 3 to use this checklist. If it was introduced in Year 3, maybe we will be more familiar with it. And now that we already have our own clinical method to approach the patient, so, sometimes difficult to put that in practice.

Student 1/Chairperson: Hmm.. hmm… somehow yes. But in this checklist, no doubt, I think there are some components, which are very useful for us, right? A bit difficult for us to adapt it, as we already have our well-established format that we have been using. So, I think this is because we are using it for the first time. That’s why we find it a bit difficult. In terms of its efficiency after you have learned to use it, how do you find the efficiency of this cheklist?

Student 4: Off hand, I can only remember one item out of the four; and that is the item ‘T’ which stands for life-threatening conditions, because that is the most important thing for us, ruling out emergency conditions. For example, when I encountered a case of antepartum hemorrhage, one of the first things I must think of is abruptio placenta because that is an emergency.

Student 2: I think the second item “W” is also very useful as it helps us to generate differential diagnoses.

Student 1/Chairperson: Yeah, yeah…I remember a patient I encountered. Every clinician said that was the case of Guillain-Barre syndrome but it turns out not to be so. Err.. it was likely because initially the visiting neurologist told every clinician at that time that the case was Guillain-Barre syndrome. But actually when I elicited the reflexes, I found it to be rather brisk. So I went back to the checklist and ask myself “what else could it be?” And I began to look hard for other evidences. Eventually after some other investigations done by the doctors in charge, it turned out that the patient actually had mononeuritis multiplex. So, I believe that there is this bias where when our superior or senior clinician says this, we may rather have the tendency to just follow the diagnosis given. What about others?

Student 3: I think as students, we are supposed to know the common conditions. So, I think, while this item “W” maybe more important for a clinician in real clinical setting, but still for us, as medical students, let’s not forget that we still need to know every common diagnoses for a particular clinical presentation.

Student 1/Chairperson: Hmm.. yeah. So, we can conclude, in terms of its efficiency, this tool does have some use to help us in formulating some of the differential diagnoses in our clinical encounters, right? OK, next, let’s move on to the aspect of the memorability of the tool. Say, after some time, you have not been using this tool. How easy it is for you to recall the items? Any thoughts? How about Student 2?

Student 2: I think for the first two items (i.e., the items “T” and “W”), they are easier for us to remember because these two items are relevant to us in our clinical encounters as medical students, because we apply them everyday. The other components or items are more difficult to remember.

Student 1/Chairperson: Okay, right. How about others?

Student 4: I don't know… I just remember the item “T”, to rule out the emergency conditions. (Student 2 nodded in agreement). The other items “E = Evidences” and “D = the Dispositional factors” do not occur to me as relevant most of the time. For example, most of the time, during bedside teaching sessions, the lecturer would straight away ask us what investigations would you likely want to do rather than asking us what evidence do you have to support your diagnosis after the history and physical examination.

Student 1/chairperson: for the item “D = the Dispositional factors” this item is not that important to us as students. Factors like emotional influences of the physician secondary to physical tiredness or sleep deprivation may not be so relevant to us because as students, we are not actually in charge of managing the patient.

Student 1: What are the errors, pitfalls, limitations or deficiencies you have encountered in using this tool?

Student 5: Perhaps the arrangement of the sequence is a bit weird. For me, it should be T-W-D-E, because after ruling out life threatening conditions and asking myself what else I want to consider, I would want to ask if there is any dispositional influences that influence my list of differential diagnoses. Only after I have finalized the list of differential diagnoses, I would ask myself to look for the evidences.

Student 4: I tend to agree Student 5. Furthermore, to look for evidences, we need time, so, it should probably be done last.

Student 2: Based on what I understand, ermm.., what I understand about ‘E = evidences’ here is not just about evidences to rule out the diagnoses but looking for evidences to support the diagnoses. As students, we are not supposed to, so much, err, looking for evidences from history and physical examination, to rule out the diagnoses, because otherwise, our lecturers might say, we are then digging our own graves when we are ordering a long list of investigations to rule out the diagnoses we gave initially.

Student 1/chairperson: yes, I tend to agree with Student 2. When we talk about ‘evidence’ in our clinical encounters, we are often talking about evidences to confirm or support the diagnoses rather than to rule out.

Student 5: In my opinion, I think after we consider ‘E = evidences’, we should go back to ‘W’, i.e., whether we are wrong or not? Or whether the evidences support my diagnoses or not? And what else it could be?

Student 1: Okay, okay. Also I think that there are some overlaps between item no. 2 (“W”) with item no. 3 (“E”). Because, by the fact that I can say I might be wrong means that I have evidences to show it to be so. So, in a way, there is some redundancy. Okay. Any other opinion on this? If not we will go to the last part, that is, on the satisfaction of using this tool. How pleasant it is to use this checklist?

Student 2: From a scale of 1 – 10 (where 10 = most satisfied), I would rate it as a 7

Student 3: To me, this tool is not pleasant to use. It is supposed to be a checklist, but to me this is too complicated. I think there are just many items in it. I cannot remember all. I think it is supposed to be simpler than this.

Student 4: I think some particular parts are quite useful; but sometimes in a stressful examination setting, I tend to forget everything except to concentrate on one particular diagnosis.

Student 3: I think we have trouble incorporating this checklist into our daily clerking

Student 4: Ahhh….as I’ve mentioned previously, because it was not introduced earlier on when we began to start our clinical years and we have our own established format of approaching the patient.

Student 2: And in stressful conditions such as in dire emergency situations, I think only the first two items are useful.

Student 1: I think even then, it is mainly it is item no. 1 that is most applicable; we think of the life threatening conditions that could occur and then we move on.

Student 5: I tend to agree with Student 3. I think the components are very difficult for me to remember. I can remember only the first 2 items.

Student 1: Hmmm… yes. Parts of this checklist are user friendly; but other parts are not. Like for example, item no. 1, the “T”. and item no. 2, “W”, “Is there any life threat?” “What if I am wrong”, the words themselves are self-explanatory. I know what it is about. But item no. 4 “dispositional factors” is complicated. I wouldn't understand what it means and I would have to read further on the fine prints to understand it. And in an emergency situation, I wouldn’t be able to do that.

Student 5: I think a checklist with 3 items would be better, rather than 4.

Student 1: Okay. So, how would you have altered the checklist if you could do so?

Student 5: As mentioned by Student 1 earlier on, there are some overlaps between Item no. 2 (“W”) and item no. 3 (“E”), so I would think item no. 3 could be removed. But, the letter “D” for “Dispositional” is quite difficult to understand, I would suggest retaining the letter “E”, but to stand for “emotion”. Even though the checklist has 2 “E”s – the environmental and emotional factor, but ultimately, it is not the environment per se that is the problem, it is the emotions generated as a result of the stressful environment that is causing the problem.

Student 1: Okay, interesting. Right, Student 4, just now you mentioned that we already have our own established format of approaching or clerking the patient. So, how different do you think this checklist is compared to the established format that you have been using?

Student 4: What we have been taught and been using is we quickly formulate our working diagnosis based on the signs and symptoms.

Student 1: Yes, it takes some time for us to learn this checklist, I mean, this is something new to us, and it is because I think many of our lecturers have seldom been emphasizing on questioning the rationale behind why we choose this working diagnosis. Alright, let’s move on. Now, student 5 mentioned that we often “forget” to use this. How else do you think we could improve on our compliance in using this checklist?

Student 5: I think we could attach this checklist in our clerking notes or clerking files. That would help to remind me on using it.

Student 1: Alright, got what you mean. I think it also very much depends on whether the respective lecturers in our previous clinical postings actually emphasized on this or not. Like for example, in ophthalmology, we are taught that the first thing you do when you approach the patient is to ask whether this condition is a sight threatening condition or not. So, until today, it sticks to my mind. When I see a patient with a visual complaint, I’ll ask whether there is any possibility of a visual threatening condition or not.

Student 1: Alright, any last words, anybody?

Student 4: I think this checklist would be useful but it should be introduced much earlier at the beginning of our clinical rotations in Year 3; then we would be more familiar with it.

Student 1: Alright, thank you. Anyone else? No? (pause). If not, thank you, everyone.
