# Supplementary material for: Perception of the usability and implementation of a metacognitive mnemonic to check cognitive errors in clinical setting
Source: BMC Med Educ. 2019 Jan 10;19:18. doi: 10.1186/s12909-018-1451-4 (PMC6327396; doi:10.1186/s12909-018-1451-4)
Supplement: Supplementary file 2 — Transcript 2 Focus Group Discussion (Group 2 final year medical students) (DOCX 16 kb) [file 12909_2018_1451_MOESM2_ESM.docx]

**Focus Group Discussion [Group 2 Final year medical students]**

Student 1(Chairperson): Hi, good afternoon everyone. Let’s start this discussion by asking a general question: how often have you used this checklist in the past 2 months or so?

Student 2: I used it occasionally, whenever I remember to use it, maybe on average, once or twice a week.

Student 3: For me, since it is a new tool, I’ve used it almost every time I see the patient.

Student 1: Okay. So, how easy do you think it is to use this checklist?

Student 6: I think this checklist is important to help us come out with differential diagnoses. To me, the item “T” is most useful.

Student 4: Yes, I agree. This checklist is basically quite similar to the way we have been taught to approach patient. But, the item “D”, to me is quite difficult to apply, as environmental or emotional influences are not really that relevant for us as students; so, I can’t relate this to the way I approach patient.

Student 1: Yes, I find the 3 items “T-W-E” to be useful; but for item “D - dispositional factors”, this is difficult for me to apply this item.

Student 3: I think the items “W” and “E” are very easy for us to apply because, “E” is what we use to confirm our provisional diagnosis and “W” is what we use to develop our differential diagnoses. Whereas, “T” is useful for us to think about the potential complications that can occur in this patient. I think “D” is a bit too difficult for me to apply as we are not in a position of treating the patient yet, and making ourselves too exhausted.

Student 5: I agree with Student 3. I think “D” is more applicable for the house officers.

Student 7: I agree too. While the checklist is applicable to us, it is helpful except for item “D”.

Student 1: What about the ease of using this checklist?

Student 3: For me personally, I find it rather difficult for me to remember the contents of the checklist initially, but once I have gone through it a few times and have understood the checklist, I think it is very easy to apply the checklist. At first, I had to refer to the checklist every time I used it but later on, I can remember the four components. It took me probably about 2 weeks to get familiarized with the checklist without referring back to it.

Student 1: I think it is also because the checklist is not that complicated to learn with only four items to it. For me, when it comes to coming up with the provisional diagnosis, I would still go for the usual flow of gathering the signs and symptoms to come up with the provisional diagnosis. I usually use the checklist towards the end of the diagnosis generation process. Because when it comes to coming up with the other differential diagnosis, then I would use the checklist to make sure that I have the evidences to support these diagnoses. What about the others? At which phase of your diagnostic approach would you use it?

Student 5: I agree, in the beginning, I find it quite difficult, but after several times, once I am familiar with it, I can use it easily.

Student 8: I use the checklist throughout the entire differential diagnosis approach. I will first ask “What else could it be? What evidences do I have to support the diagnosis I have in mind?” And I will end by applying the “T” – what other life threatening conditions that I might have missed?

Student 1: Okay. How often have you used the checklist in the past one month or so? I used it mostly for every case. How about others? And what do you think of its memorability?

Student 8: Honestly, when I first used it, I can only remember the item T, which is “Threat”. It’s only after awhile, I am more familiar with it but still, I can’t remember all the items.

Student 1: What about you, Student 7? How useful do you find this checklist?

Student 7: I have ever encountered a case of a patient presented with acute epigastric pain. At that time, I thought I was very sure it was a case of acute pancreatitis. However, when I reflected on the evidences to support the diagnosis of acute pancreatitis, I realized that I was wrong. It was actually a case of acute peptic ulcer disease. But at that time, my mind was narrowed to the diagnosis of acute pancreatitis only. So I think this checklist is really helpful in reminding us to be more broad-minded.

Student 1: Student 3, what do you think of some of the flaws or pitfalls in using this checklist?

Student 1: I believe the challenge in using this checklist is contributed by our own lack of knowledge. It is mostly because of our own inadequacies; for example, we can usually only think of 3 - 4 differential diagnoses. And as medical students, we are very contented with those 3 – 4 differential diagnoses. But when we refer to the specialist or the lecturer, then we are being pointed out to what we have missed. Which means that, to even use this checklist, we need to have enough background knowledge.

Student 1: Yes, in a practical sense, I think this checklist is more applicable for house officers and doctors whereas for us as medical students, as our aim is to first pass exam, our focus would then be to generate a list of differential diagnosis for that particular discipline itself. For example, if we are seeing a patient in surgery discipline, our differential diagnosis would confined to that of surgery, and we probably won’t think of causes in internal medicine or pediatric or obstetrics and gynecology. This checklist would be more useful when we start our internship. Alright. Let’s move on. How satisfied or how pleasant in using this checklist?

Student 3: Actually I think this checklist is not very pleasant to use in the sense that it reminds me of my own inadequacies and shortcomings but it is still a good checklist to use to help us remember to check for things we might have missed.

Student 1: I think the checklist is a good add-on to the clinical approach that we have been taught. For example, once I was seeing a patient with epigastric pain. Because of her relatively young age, the diagnosis of cancer didn't readily cross my mind initially. I was thinking of peptic ulcer disease. However, as I applied the checklist (especially on the item “W – what if I am wrong?”, I realize that it cannot be just peptic ulcer disease. Eventually, it turns out to be gastric carcinoma.

Student 4: I agree with Student 1 that this checklist is a useful adjunct. In case if we forget certain important elements in the history, this checklist serves as a reminder for us to ask those relevant questions. But as others have mentioned, I think the item “D” not useful at this stage as a medical student.

Student 5: I also agree with that. I think it is pleasant to use this checklist with its mnemonic structure and also because, since it only has four items, it is simple enough. I usually prefer to use it at the end of the history taking and physical examination.

Student 3: Well, contrary to what others might have said, I think the item “D” is still relevant to us as medical students. Since we are humans, our judgment can also be influenced by the emotional state that we are in. But the real problem is, I think, even if we know the emotional and environment dispositions that may influence our judgment, often we are still not able to generate alternative diagnoses due to the lack of knowledge.

Student 1: Anyone wants to raise any more points for discussion? (pause) If not, that’s it. Thank you.
