# Supplementary material for: Perception of the usability and implementation of a metacognitive mnemonic to check cognitive errors in clinical setting
Source: BMC Med Educ. 2019 Jan 10;19:18. doi: 10.1186/s12909-018-1451-4 (PMC6327396; doi:10.1186/s12909-018-1451-4)
Supplement: Supplementary file 3 — Transcript 3 Focus Groupd Discussion (Doctors Group) (DOCX 24 kb) [file 12909_2018_1451_MOESM3_ESM.docx]

**Focus Group Discussion [Doctors Group]**

Doctor 4 (Chairperson): Good morning, everyone. Let’s start our discussion by looking at the usability of this checklist. Anyone would like to start?

Doctor 3: Hi. For me, I think subconsciously, I already have been reflecting on the items in this checklist all along. But when I am encountering a challenging case, I think having this tool at hand is particularly useful particularly when it is due to dilemma in diagnosis.

Doctor 4 (Chairperson): Ok. Doctor 11, at what stage during the diagnostic process would you use this tool?

Doctor 11: I think I use it more for life threatening emergency cases in the “red zone”^[[1]](#footnote-1)^ (resuscitation zone), particularly when the patient is not improving.

Doctor 2: Hi. For me, I usually use this tool when I am screening patients at the secondary triage area^[[2]](#footnote-2)^. This is because this is the area where I would discharge most of the patients, and of course, this is also the area where I would admit most of the “Green zone” or “Yellow zone” patients. The learnability of this tool is quite easy because it has a mnemonic to it. Here, it is helpful to have a quick first look by asking whether if there is any potential life or limb threat in this patient or if the patient is in a shock condition. This is where the item “T” of the checklist comes in very handy in secondary triage. Then when taking history to come up with the differential diagnoses, the item “W” becomes very handy particularly for patients who return for second visit or more. I would ask “what if I am wrong?” “What else could it be?”. In terms of coming up with the evidences, hmm…, this can be a bit tricky. It depends. It depends on how much knowledge do I have. If my knowledge on a particular disease is not that great, then I might not be able to come up with a lot of reasons to support my diagnosis. For emotional influences, it depends. If I have a lot of patients that I have to attend to, I may tend to get frustrated and this may affect my judgment. So, yes, the tool is easy to use, it helps us to be more careful with our diagnoses but it also very much dependent on our depth of knowledge.

Doctor 8: For me, I think the tool is more useful in managing polytrauma patients. This is because, for polytrauma patients, especially when they are unstable, we try to stabilize them first. But at times, we may miss some hidden injuries, additional injuries, etc. This tool helps us to keep reminding ourselves so as not to miss other hidden diagnoses.

Doctor 9: I feel that this tool is very helpful in evaluating patients in the Green zone. This is because of the volume of the patients that come in to Green zone, especially for patients that come in with very nonspecific complaints. So, by just applying this checklist, it can help us to rule out the life threatening causes for these nonspecific complaints, and as long as we can rule out the life threatening causes even for these nonspecific complaints, we can safely discharge them.

Doctor 4: Do you think it is helpful to teach this tool to our house officers?

Doctor 1: Yes, I guess it would be useful but I think the house officers who come to the emergency department for their clinical rotation are those in the last or second last rotation. These house officers are considered the senior house officers and by then, I think, just like us, subconsciously, they would already have developed their own approaches to clinical diagnosis. Unless, if they are being introduced to this tool earlier in the first few rotations.

Doctor 4: Okay. Any other opinion?

Doctor 6: I suppose, if I have learned this earlier when I was a junior doctor, I might have been more confident in patient management, and might have a lesser risk of missing out important diagnosis. I think, I would have appreciated this if I have known about this a bit younger in my career path.

Doctor 10: Yeah, this tool should be taught earlier... as early as possible, maybe while in medical school because once they already out working, the junior doctors would have already developed their own ways of approaching patients, and it might be difficult to introduce new tools for them. Whereas, if we have taught them about this tool much earlier, they would have started to use it much earlier, and it will become a good habit for them.

Doctor 4: Regarding this tool, do you think it is easy to remember the components of this tool?

Doctor 5: I think generally the contents of this tool are exactly what we do in our day-to-day clinical works. Basically when we see patients, no matter which zones we are working in, the first thing is to rule out life threatening causes; only then we start ruling out all other possible diagnoses, and only then we make decisions on whether to admit or to discharge the patients. So, basically, thought processes embedded in the tool is very simple and generic, and it is applicable for every doctor who practices. So…, I think it is very easy to remember although its sequence may be “jumbled up” a bit with our individual thought process, but basically that’s what we do when we see patients.

Doctor 9: Basically I think it is applicable to us, as we are dealing with patients in the emergency department. So, it is our “bread and butter” and we are doing it subconsciously, but observing how junior doctors work, this might be a bit new for them as they are in the transition from the conventional way of approaching patient. Their approach is different and ours here is on stabilizing the patient, approaching fresh new case whom you are not sure what you are dealing with at that particular point. So, I think this might be something new for these junior doctors and it is a good thing to expose them early so that it gets into their subconscious minds.

Doctor 4: Have you encountered any pitfalls in using this tool thus far?

Doctor 6: So far, I have encountered any pitfall although when I am very busy I tend to forget to use it.

Doctor 7: Hmmm, similarly I haven’t encountered any pitfall yet and in fact, particularly with regards to the “E = environment” influence, I have a story to share. I think when the environment become uncontrollably busy, like when you have a number of patients to manage simultaneously, there may be certain patients that I do not pay enough attention to. There was once when I had to manage 3 patients with altered mental status at a go, there was one patient that arrived way earlier with low blood pressure, tachycardic but not tachypneic... and I could not figure out what was wrong with him until hours later when I found out that the patient had chest pain, only then my diagnosis changed from septic shock to acute pulmonary embolism. The reason why I missed the chest pain history was because at that time of the busy environment, all my attention was focused on another patient with altered mental status. I did not really pay attention to another patient until he complained of the worsening chest pain.

Doctor 8: To me the limitation of this tool is that when we keep thinking too much on the patient, we may then be worrying too much about the case, spending too much time thinking of what could the errors be, etc. I mean, being a bit skeptical, applying critical thinking is good, but sometimes, this can become too time-consuming especially when we are too skeptical, which in turn, delays our management, leading to stress and frustration and prolonging the waiting time for the patients. This, I think is particularly true for cases that are stable or relatively stable; in Green zone, for example, although, I mean, this tool would be helpful for patients with unstable vital signs, but for patients who are stable in Green zone, I think, applying this tool is too time consuming. In other words, where the diagnosis is clear cut, I would probably not likely to apply the tool, but where the diagnosis is not clear cut, but I know something is not right the patient, I would probably apply it. The challenge for us then is to know when and for which case do we need to apply the checklist, and which ones we do not.

Doctor 4: Any opinion? [pause] If no, let’s move on. So far, how satisfied are you in using this tool in your daily practice?

Doctor 10: Yes, definitely. This tool will at least helps me not to simply discharge the the patient, reducing my risk of misdiagnosing and mismanaging the patient.

Doctor 3: Basically I am quite satisfied with using this tool. This is especially so for managing a psychiatric patient because often times, we may have some biases towards this group of patient, dismissing their complaints or assuming that their symptoms as something not genuine.

Doctor 9: For me, I think the components “T”, “W”, “E” are something which we are already practicing on the daily basis even without referring to the checklist but the “D = Disposition” component of the tool is something we need to pay particular attention too. The “environment” we are working in can influence our judgment. For example, I think, many of us are put off by patients or their relatives who start asking for sick leave within minutes of the interview process. I think that this can cloud our judgment. Just for example, yesterday I encountered a patient in green zone, after just 2 questions, the relatives of the patient suddenly asked me whether I could provide a sick leave for the following day or not. At that point, I felt that it could cloud my judgment. So, I immediately tried to remove that distraction. I asked the relatives to wait outside of the consultation room while I managed the patient. It was only after the relatives had stayed out, I noticed that the blood pressure of the patient was high and he was only 18 years old. If I did not eliminate the distraction, I might have missed the high blood pressure in an 18-year old patient.

Doctor 4: Anyone else with similar experiences?

Doctor 10: I had a similar near-miss encounter before. It was an 11-year boy, apparently healthy with no previous medical illnesses. He developed a “sharp pricking” kind of pain on his left knee for 2 weeks’ duration following a hit by a football. But according to patient, the ball only hit his knee lightly. But the parents were actually quite curious why the pain did not resolve but keeps coming back. But there was no swelling and no surrounding skin changes. He looked perfectly well, able to ambulate and so on. So, initially I thought of discharging this patient who looked so well but ehh… at that time, I applied this tool and asked myself what else I could have missed given the unusual presentation of left knee pain. Furthermore, the parents were quite worried. So, I decided to do an x-ray of for him and lo and behold, I’ve found out that this pain is due osteochondroma. So, if I had not found out the cause for him, the family members might have brought the child to go and “shop” for another medical practitioner from place to place.

Doctor 5: I just had another similar case yesterday while working at the secondary triage. This patient was a small child, a preschooler, who was brought in by the parents because of an alleged fall with the complaint that the child was initially not moving the affected limb after the fall. But subsequently, the child was able to move the limb again, and he was very active and well. And when I examined the child, the child seems well and comfortable, he was active, not crying and not irritable. There was no swelling, no redness of skin and the range of movement of that limb was full. Furthermore, the child was actually quite chubby, thus I was a bit concerned whether that could have made my examination findings less reliable. I was planning to discharge the child but I just felt that I need more evidences to support my decision and to assure the parents that their child is well enough to go home. So, I decided to get an X-ray for the child but lo and behold, the X-ray actually showed that the child had a chip fracture of the olecranon. So, reflecting on this tool can be very helpful in this sense.

Doctor 4: Anyone else with any experience to share?

Doctor 2: I have another case here to share. I was working in the secondary triage area when I saw a patient who is mentally disabled, brought in by the parents that the child had cough and fever for one day duration. But other than that, he looked quite well. His vital signs were stable. My impression at that time was URTI. Just as I was about to discharge the patient, out of curiosity, I asked myself, why was the patient on wheelchair? He did not need to be on wheelchair if he just had URTI. How long has he been on wheelchair? Is it because he’s not able to walk by himself? And if he’s not able to walk, how long has he been in this state? Then I asked myself, “What else could this be then?” And then I asked the family members, “Why is he on a wheelchair? Has he always been on wheelchair, not able to walk on his own?” It was only then that the family member said, “Oh, I am sorry. I forgot to inform you just now. He suddenly cannot walked. Just about 2 hours ago.” And there it is. To cut the long story short, he was subsequently diagnosed with a stroke, and thrombolysis was given. From a mere URTI to a full blown stroke! So, well, yes, history is important, and it is important to observe the patient well, but sometimes, having a mind of curiosity is just so important.

Doctor 4: Anybody else has anything to share?

Doctor 1: My experience was also in secondary triage. I think most of our experiences happened in secondary triage area. So, I think this tool is particularly useful when you are working in secondary triage, when you have to make a decision whether to keep or to discharge the patient. My case just happened last week. I had a patient diagnosed with left lower limb cellulitis for more than a week already. Earlier on the same day when she came to the emergency department, that same morning, she had already sought consultation in a general practitioner clinic and had already been started on a course of antibiotic. And she had also been given an appointment to get an x-ray done in a private medical center. The x-ray was taken already in fact; but later on when they went back home, some of their family members and friends commented that probably they should get a second opinion; hmm… saying things like, “maybe it is not a cellulitis after all” etc. And that actually caused the patient to worry quite a bit. When I first saw the patient, my first impression was like, okay, you already have this for a week and you’ve already seen a doctor, and you’ve just started been started on a course of antibiotic, so, you probably don’t need a second opinion so quick. And, you have just had an x-ray done which was reported as normal, and so, probably not going to repeat another x-ray, exposing you to more radiation. So, initially I suggested to them to just continue with the antibiotic and come back few days later if not responding to the treatment. But you know what, I was thinking to myself, since they are here, I might as well get more assurance, and probably just to rule out any other possible diagnosis [33]

Doctor 4: Alright for all of you who have shared your stories, how exactly did you use it? Do you have to consciously or deliberately use it, step-by-step, quadrant-by-quadrant, or is it that the contents of the tool are already in your subconscious thought process? And if you have already automatized or embedded the contents of the tool as part of your clinical approach, how long do you think a junior doctor would take to automatize the contents of this tool?

Doctor 8: I think as most of us already have more than one year of experience working in the emergency department, we should be able to incorporate the items of this tool subconsciously. It is just that by having this tool, with the contents of this tool lined up in 4 quadrants, it becomes more organized and systematic. So, it is not difficult for us to incorporate this tool. But I think it is good to implement it for all junior doctors, including those in the primary care because they tend to make mistakes. Primary care is very important.

Doctor 9: Even before becoming a medical officer, I remembered I had a senior who reminded me always that, whenever I have dilemma in diagnosing a condition, the first thing to do is to rule out life threatening conditions. So, I think the most important thing is to make ourselves aware of all these things.

Doctor 3: I think the D = dispositional influence is very important especially for us as medical doctors doing shift work in the emergency department. And so, especially during night shift, when you can be rather tired after a busy night, our sleeping pattern, our circardian rhythm can be disrupted. I had a case previously, which is a case of a middle-aged male patient with history of hypertension, came to the emergency department at night, with symptoms suggestive of renal colic. But on top of that, this patient also has history of end stage renal failure. So at that point of assessment, I noticed that the blood pressure dropped slightly after given some opioid. So, initially we attributed the drop in blood pressure to the adverse effect of the opioid. But we observed the patient over night as we were tired after a long busy night. We kept the patient, not making any decision whether to admit or to discharge until the following morning. The following morning, we handed over the case to the morning shift doctor, and only then it was found that the patient had abdominal aneurysm, although there wasn’t any obvious abdominal pulsatile mass on physical examination. So the lesson is, we need to be aware of our own limitations, unless it is absolutely necessary to make urgent or emergency decisions, it is best to defer until we are in a better state of mind. Yeah, it may not be that wise to make rather big decisions when we are fatigue.

Doctor 4: Yeah, that’s right. Thanks for the input. Anybody else with anything to share? [pause] Okay, if no more, thank you very much for your participation.

1. Red zone refers to the zone where the patients have life or limb threatening or potentially threatening conditions that requiring immediate management and/or critical interventions. Yellow zone is the zone where the patients are in semi-critical conditions although these patients do not have immediate life or limb threat. Green zone is the zone where the patients are hemodynamically stable but they require admission for in-hospital management. [↑](#footnote-ref-1)
2. In the participants’ hospital (Sarawak General Hospital), secondary triage is the area in the emergency department where the medical doctor would screen the patient for the second time in order to decide whether the patient would be stable enough for discharge (for minor conditions) or has to be admitted for further investigations and treatment, and to which zones the patient would be assigned to (often either to Green zone or Yellow zone; although occasionally patients requiring resuscitative measures might have been missed in the prior primary triage area). Secondary triage is also the area where the doctor would decide and order any necessary bedside investigations (such as electrocardiography) or radiological imaging studies that may have a bearing on the patient’s immediate management. Primary triage area is the first contact of the patient with the healthcare staff and is usually handled by the paramedics and nurses. In the primary triage, the patient will first be screened whether he or she is stable to be screened further in the secondary triage area by the doctor or to be brought in to the resuscitative zone (also known as Red zone) immediately. [↑](#footnote-ref-2)
